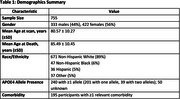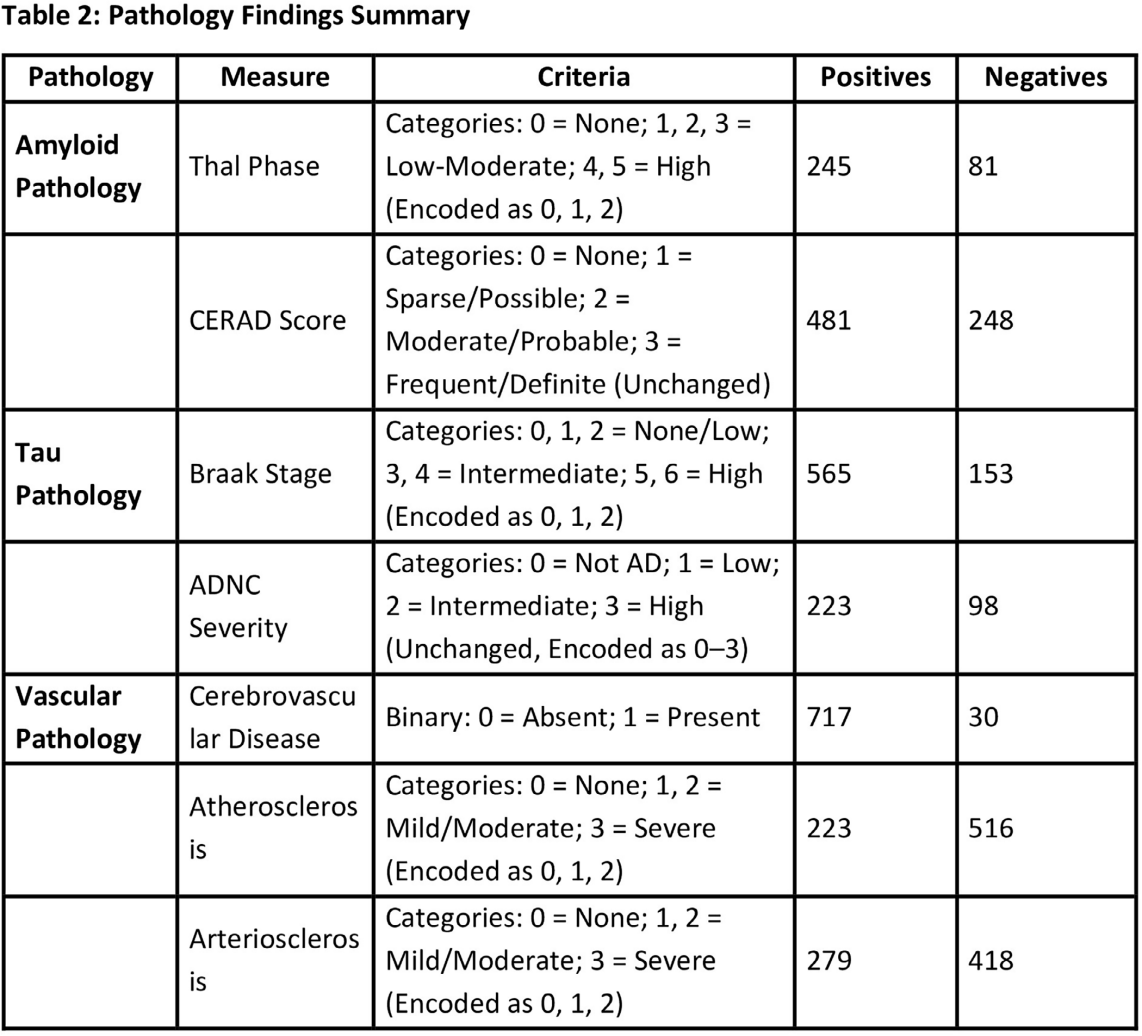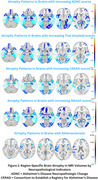# Bridging Neuroimaging and Pathology in Dementia: A Multi‐Cohort Investigation of MRI‐Derived Brain Volumes

**DOI:** 10.1002/alz70856_105854

**Published:** 2026-01-09

**Authors:** Rajeswar Kumar, Tanweer Rashid, Sokratis Charisis, Karl Li, Ngoc‐Huynh Ho, Sachintha Ransara Brandigampala, Arthur W. Toga, Walter W. Kukull, Shannon L Risacher, Di Wang, Mariam Mojtabai, Nicolas Honnorat, Derek B. Archer, Duygu Tosun, David H Wang, David A. A. Bennett, David M Martinez, Angela L. Jefferson, Susan M. Resnick, Andrew J. Saykin, Sudha Seshadri, Michael L Cuccaro, Christos Davatzikos, Timothy J. Hohman, Mohamad Habes, The Alzheimer's Disease Sequencing Project (ADSP)

**Affiliations:** ^1^ Glenn Biggs Institute for Alzheimer's & Neurodegenerative Diseases, University of Texas Health Sciences Center at San Antonio, San Antonio, TX, USA; ^2^ University of Texas Health Science Center at San Antonio, San Antonio, TX, USA; ^3^ Laboratory of Neuro Imaging, Stevens Neuroimaging and Informatics Institute, Keck School of Medicine, University of Southern California, Los Angeles, CA, USA; ^4^ National Alzheimer's Coordinating Center, University of Washington, Seattle, WA, USA; ^5^ Department of Radiology and Imaging Sciences, Indiana University School of Medicine, Indianapolis, IN, USA; ^6^ Indiana Alzheimer's Disease Research Center, Indianapolis, IN, USA; ^7^ UT Health Science Center at San Antonio, San Antonio, TX, USA; ^8^ Glenn Biggs Institute for Alzheimer's & Neurodegenerative Diseases, University of Texas Health Sciences Center at San Antonio, San Antonio, TX, USA; ^9^ Vanderbilt Genetics Institute, Vanderbilt University Medical Center, Nashville, TN, USA; ^10^ Vanderbilt Memory & Alzheimer's Center, Vanderbilt University Medical Center, Nashville, TN, USA; ^11^ Vanderbilt Brain Institute, Vanderbilt University Medical Center, Nashville, TN, USA; ^12^ Department of Neurology, Vanderbilt Memory & Alzheimer's Center, Vanderbilt University Medical Center, Nashville, TN, USA; ^13^ Department of Radiology and Biomedical Imaging, University of California, San Francisco, San Francisco, CA, USA; ^14^ Rush Alzheimer's Disease Center, Rush University Medical Center, Chicago, IL, USA; ^15^ Vanderbilt Memory and Alzheimer's Center, Vanderbilt University School of Medicine, Nashville, TN, USA; ^16^ Department of Computer Science, Vanderbilt University, Nashville, TN, USA; ^17^ Laboratory of Behavioral Neuroscience, National Institute on Aging Intramural Research Program, National Institutes of Health, Baltimore, MD, USA; ^18^ Indiana Alzheimer's Disease Research Center, Indiana University School of Medicine, Indianapolis, IN, USA; ^19^ Glenn Biggs Institute for Alzheimer's & Neurodegenerative Diseases, University of Texas Health Science Center, San Antonio, TX, USA; ^20^ Dr. John T. Macdonald Foundation Department of Human Genetics, University of Miami Miller School of Medicine, Miami, FL, USA; ^21^ The John P. Hussman Institute for Human Genomics, University of Miami, Miami, FL, USA; ^22^ Department of Radiology, University of Pennsylvania, Philadelphia, PA, USA; ^23^ Department of Neurology, Vanderbilt University Medical Center, Nashville, TN, USA

## Abstract

**Background:**

The ability to precisely characterize neurodegenerative diseases at early stages remains a challenge. Understanding the relationship between magnetic resonance Imaging (MRI)‐derived brain volumes and neuropathological outcomes is key to advancing early and accurate diagnosis of neurodegenerative diseases. While MRI‐based volumetrics are widely used to assess structural changes in vivo, their link to neuropathological findings remains underexplored. This study examines associations between region‐specific brain atrophy and neuropathological markers, including Alzheimer's Disease Neuropathologic Change (ADNC), CERAD score, Braak staging, and Thal amyloid phase, leveraging volumetric data across multiple aging cohorts.

**Method:**

T1‐weighted MRI scans from participants in longitudinal dementia cohorts (ROS, MAP, MARS, and NACC) were pre‐processed, and brain volumes of 120 cerebral regions of interest (ROIs) were calculated using Multi‐Atlas Segmentation Utilizing Ensembles (MUSE). Associations between ROI volumes and neuropathological metrics were analysed using linear regression, adjusting for age at scan, difference between age at death and age at scan, sex, intracranial volume, and site/scanner. Neuropathological metrics, including ADNC, CERAD scores, Thal phase, Braak staging, and vascular metrics like atherosclerosis were analysed as continuous variables grouped categorically (e.g., Thal phase recoded into three categories: 0, 1, and 2). Statistical significance was determined using Benjamini‐Hochberg method (*p* <0.05).

**Result:**

Among 755 participants (mean age at scan: 80.57±10.23 years; mean age at death: 85.49±10.45 years; 44% male), ADNC was associated with atrophy in temporal and parietal regions, notably middle and inferior temporal gyri, angular gyrus, and amygdala. CERAD scores correlated with atrophy in middle temporal, para‐hippocampal, and fusiform gyrus, Thal phase with atrophy in middle temporal, angular gyrus, and precuneus and Braak staging showed atrophy in entorhinal cortex, middle temporal gyrus, and para‐hippocampal regions. Atherosclerosis was associated with atrophy in prefrontal cortex, and parieto‐occipital regions.

**Conclusion:**

These findings highlight the potential of MRI‐derived volumetrics as non‐invasive biomarkers of underlying Alzheimer's pathology. Our findings demonstrate notable atrophy in medial temporal, lateral temporoparietal, and midline parietal regions, supporting recognized AD patterns of neurodegeneration, consistent with pivotal studies by Habes et al.,(2016), Dickerson et al.,(2009). Associations between amyloid, tau burdens and atrophy in temporal, parietal, and limbic regions underscore their diagnostic value in neurodegeneration.